# DREAM—Meta learning for tailored hierarchical dialogue management: application to motivational interview

**DOI:** 10.3389/frai.2026.1786834

**Published:** 2026-06-24

**Authors:** Lucie Galland, Catherine Pelachaud, Florian Pecune

**Affiliations:** 1 ISIR, Sorbonne University, Paris, France; 2 CNRS, Paris, France; 3 SANPSY, University of Bordeaux, Bordeaux, France

**Keywords:** adaptation, dialogue management, LLM, motivational interview, personalization

## Abstract

Large Language Models (LLM) enable open-ended and socially rich interactions, yet they lack mechanisms for deliberate control and structured adaptation. In many complex tasks, effective dialogue requires two levels of adaptation: personalization to diverse user profiles with distinct needs, and dynamic management of evolving sub-goals throughout multi-stage interactions. Existing systems typically address only one of these dimensions, limiting their ability to support nuanced, goal-driven conversations. We propose a hybrid dialogue framework that integrates an LLM with a hierarchical dialogue manager capable of guiding conversational structure while adapting to different user types. Motivational Interviewing (MI) serves as a challenging testbed, as it requires both adherence to a defined sequence of interaction phases and sensitive personalization to individual users. Our manager leverages hierarchical reinforcement learning to model MI's multi-phase progression and employs meta-learning to enable rapid adaptation across user profiles. Experiments using a simulated user and evaluations involving human participants show that the proposed system produces more engaging interactions than a plain LLM baseline. This article demonstrates the value of hybrid architectures for achieving two-level adaptation in dialogue systems.

## 1 Introduction

Recent advances in natural language processing have enabled the emergence of large language models (LLMs) capable of supporting open-ended, free-form interactions across a wide range of tasks. In contrast to earlier rule-based dialogue systems, which offered strong controllability but limited flexibility (Shidara et al., 2020), LLMs now allow users to converse without predefined scripts or rigid dialogue flows. This increased naturalness has raised expectations: users now anticipate conversational systems that are not only fluent but also adaptive, responsive, and able to personalize their behavior across contexts and tasks (Yi et al., 2024; Cambria et al., 2024).

However, the adaptability offered by LLMs is mainly limited to imitation. Their flexibility stems from pattern replication; LLMs generalize from vast datasets and can mimic many linguistic forms, yet they do not actively tailor their behavior to different user profiles (Boisseau, 2024). In practice, users may vary widely in terms of needs, communication styles, and levels of prior knowledge (e.g., beginners vs. experts, hesitant users vs. highly motivated users), and supporting such diversity requires genuine personalization in addition to imitation.

A second limitation concerns dialogue phase adaptation. Dialogue is commonly understood as being separated into distinct phases. Each dialogue typically includes at least an “Opening phase” and a “Closing phase”, which frame the interaction, and the “Body phase”, where the main content or task is handled (Mondada, 2025). In more complex dialogues, the Body phase can itself be subdivided into sub-goals according to the task: for instance, a virtual teacher might first determine the level of the student, then move into a learning module (Macina et al., 2023); in a medical consultation, the practitioner may gather patient information, then perform diagnosis/planning (Miller et al., 2003). While rule-based or planner-based systems traditionally handle such structure explicitly, LLMs often focus narrowly on the immediate prompt and the overarching goal. As a result, they struggle to maintain long-term coherence, to track sub-objectives, or to decide when and how to transition between phases of a complex task (He et al., 2024).

These two forms of adaptation: adapting to user profiles and adapting to dialogue phases and subgoals are critical for domains such as education (Mogavi et al., 2024), coaching (Steenstra et al., 2024), mental health support (Martin and Richmond, 2023), etc. A promising solution lies in hybrid architectures that guide LLMs through explicit control mechanisms. By combining the generative strengths of LLMs with a dedicated dialogue manager, such models can enforce structure and controllability while preserving naturalness. Such an approach has been explored in Galland et al. (2024), showing that the hybrid method can bring controllability while improving naturalness. In this work, we introduce a hybrid system specifically designed to adapt to user profiles and different dialogue phases. We apply this framework to Motivational Interviews (MI), a style of communication with the goal to motivate a user to change an unhealthy behavior (Miller and Rollnick, 2012).

Our contributions are threefold:

- We develop a reinforcement-learning-based dialogue manager capable of adapting both to user profiles and to different dialogue phases, enabling two-level adaptation essential for therapeutic dialogue.
- We integrate this dialogue manager with an LLM, creating a hybrid agent that combines structured decision-making with flexible language generation.
- We evaluate the system with human participants, demonstrating that our hybrid framework improves adaptation to both user profiles and dialogue phases over a plain LLM for our MI application.

The remainder of this study is organized as follows: Section 2 discusses related work; Section 3 introduces the theoretical background on our application case, MI; Section 4 presents our methodology; Section 5 details the evaluation environment; Section 6 reports the training results; Section 7 analyzes and interprets these findings; and Section 8 describes the human-subject evaluation presented in details in Galland et al. (2025b).

## 2 Related work

Recent advances in adaptive dialogue systems lie at the intersection of three key capabilities: open-ended, free-form dialogue generation, adaptation to diverse user profiles, and adaptation to evolving sub-goals or phases within a task. While many existing systems achieve one or sometimes two of these components, no prior framework fully integrates all three. In this section, we review the literature along each axis and highlight the resulting gap, which our study aims to address.

### 2.1 Free-form dialogue generation with large language models

LLMs have fundamentally altered the paradigm of dialogue generation. Survey papers such as Yi et al. (2024) and Cambria et al. (2024) document the transition from rule-based and modular systems toward LLM-driven architectures capable of managing unstructured, natural conversation. GPT-like models demonstrate strong generative performance across diverse tasks (Baktash and Dawodi, 2023) and have been shown to support open-domain, multi-turn interactions (Chu et al., 2024). This shift has empowered conversational agents with unprecedented expressive flexibility.

However, LLMs suffer from well-known limitations: hallucinations (Chu et al., 2024) or reduced long-term coherence (He et al., 2024). Recent works propose solutions such as contextual planning (He et al., 2024), or interactive agent modeling (Zhang et al., 2020), but these approaches rarely incorporate structured decision-making.

### 2.2 Adaptation to User Profiles

Adapting dialogue to individual differences, such as preferences, goals, emotional states, or social behaviors, is a long-standing challenge in intelligent interactive systems.

In dialogue research, several systems aim to personalize conversational behavior. Kola et al. (2023) shows that psychological characteristics can be successfully used as a basis for explanations given to users about the decisions of an agenda management personal assistant agent, Greco et al. (2023) shows that adapting dialogue to users' knowledge level can improve task accuracy, and Pecune et al. (2022) shows that personalized recommendations improve perception of an agent. Hybrid state trackers (Xu et al., 2023), statistical user models (Weber et al., 2018; Galland et al., 2022), multi-policy learning (Takanobu et al., 2020), and meta-learning strategies (Zhang et al., 2020) have been employed to adapt to different user types. Similarly, retrieval-augmented LLMs (Pattnayak et al., 2025) and persona-conditioned models attempt to improve personalization. Reinforcement learning RL method can also be used for user adaptation (Walker, 2000). Advanced RL frameworks introduce social rewards (Pecune and Marsella, 2020) or dynamic reward shaping (Weber et al., 2018) to adapt to user profiles.

Nevertheless, these approaches rarely combine personalization with explicit management of task structure or dynamic sub-goal sequencing. Moreover, many focus on short-term user signals and fail to incorporate long-horizon adaptation across conversation phases.

### 2.3 Adaptation to sub-goals and dialogue phases

Beyond personalization, many applications require agents to manage structured sequences of sub-goals or phases–for example, multi-step assistance, tutoring dialogues, healthcare consultations, or negotiation tasks. Rule-based dialogue systems traditionally excel in such structured settings (Shidara et al., 2020). RL has been used to optimize phase-level dialogue policies (Li et al., 2016; Takanobu et al., 2020).

Recent work attempts to integrate LLMs with planning or structured decision-making. Prompt optimization with RL (Yao et al., 2023), tree-search control over LLM decisions (Yu et al., 2023), or dynamic routing architectures (Pattnayak et al., 2025) have improved behavior consistency in complex tasks. Williams and Young (2007) have worked on scaling POMDP to handle more complex dialogues. Some studies have explored training dialogue systems with RL and simulated user (Rieser et al., 2010), also showing that policies can be transferred to real users (Lemon et al., 2006). More advanced frameworks have incorporated hierarchical reinforcement learning to handle subgoals in task dialogue (Cuayáhuitl et al., 2010). However, these systems generally do not jointly address personalized user adaptation.

### 2.4 Hybrid approaches: toward more control and adaptation in LLM systems

Hybrid models combining symbolic, statistical, and neural components aim to balance the control of traditional systems with the flexibility of LLMs. Recent frameworks integrate smaller fine-tuned models with larger general-purpose LLMs (Xu et al., 2023), utilize RL to guide contextual decisions (Ismail et al., 2022), or include intent recognition and dialog act planning layers (Griol and Callejas, 2025). These works suggest that using a hybrid framework with a dialogue manager controlling an LLM can improve dialogue.

Despite these advances, none of these approaches simultaneously achieve: at the same time, free-form, natural LLM-based dialogue, adaptation to heterogeneous user profiles, and structured adaptation to dynamic sub-goals or phases.

Most systems offer generative freedom or structured control, but not both; or they personalize interactions without managing multi-phase task progression.

Most of the existing systems typically cover only a subset of the three capabilities:

- LLM-based systems excel at free-form dialogue, but lack structured control and deep personalization.
- Cognitive and user-modeling approaches provide user-profile adaptation, but seldom integrate LLM-level generative flexibility.
- RL-based and rule-based dialogue managers offer sub-goal / phase adaptation, but, so far, rely on fixed templates and limited linguistic diversity.

To the best of our knowledge, no prior study has proposed a unified architecture that integrates all three dimensions within a single framework. Current state-of-the-art dialogue systems for tasks such as MI, which necessitate adaptation to diverse user profiles as well as sequential and embedded phases, rely either on rule-based models (Prochaska et al., 2021) or plain LLMs (Steenstra et al., 2024). A rule-based model necessitates the drafting of carefully crafted rules. In this work, we investigate whether the current state-of-the-art baseline for MI dialogue (the plain LLM) can be significantly improved by incorporating a model that guides the selection of dialogue acts.

This gap motivates our proposed approach: a hybrid RL-based dialogue manager coupled with an LLM. This architecture is capable of managing conversational phases and adapting to diverse user profiles while simultaneously preserving the fluidity of natural, free-form dialogue.

## 3 Application: motivational interviewing

MI is a therapeutic approach that emphasizes collaboration and supports behavioral change by guiding patients to explore the reasons and motivations behind their unhealthy behaviors.

### 3.1 Dialogue with multiple phases

**Figure 1:**
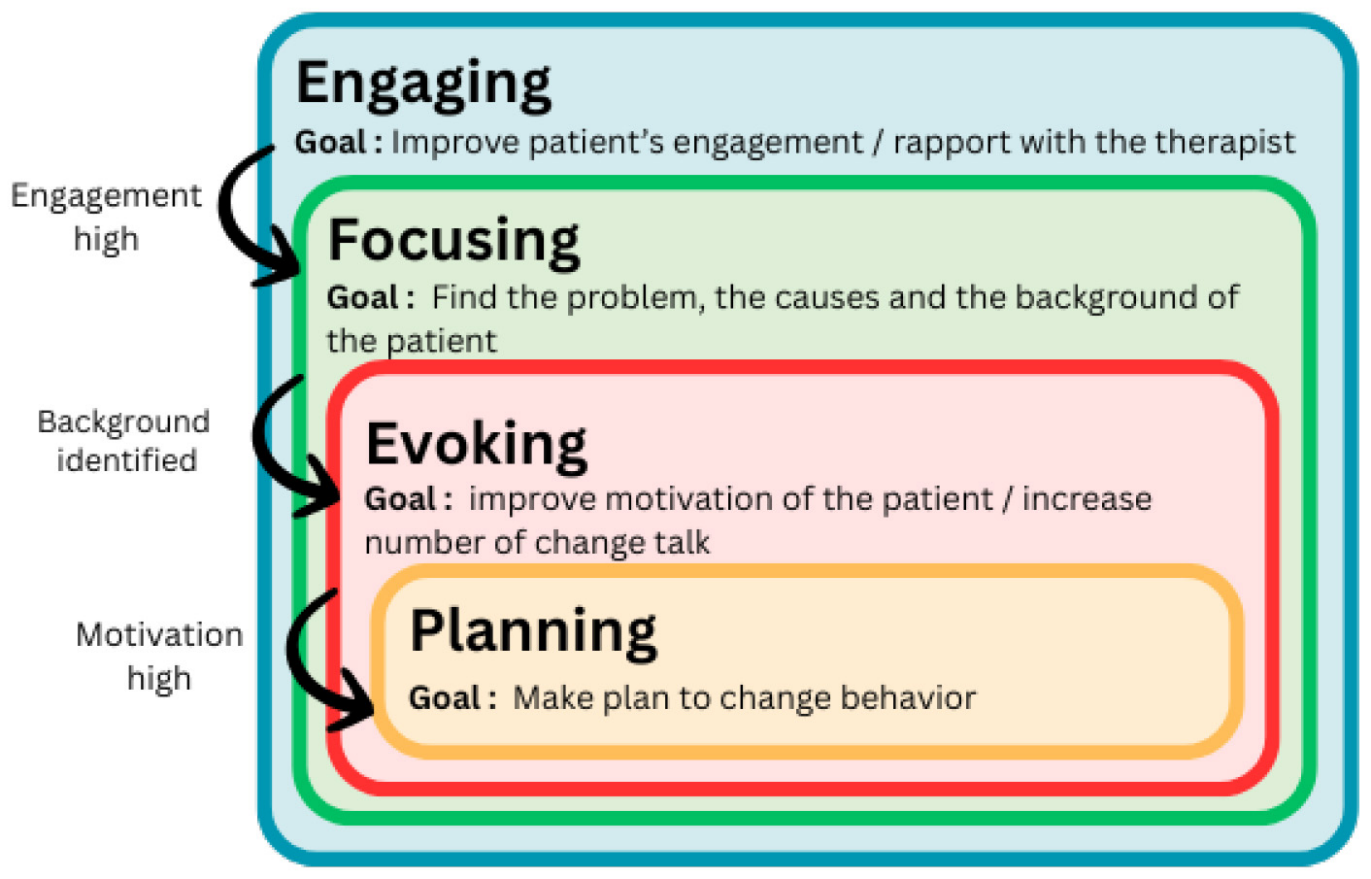
Phases of motivational interviewing.

Complex dialogues such as MI dialogues are composed of distinct phases, each governed by specific long-term strategies (Miller and Rollnick, 2012) (see Figure 1). MI dialogues typically begin with an *Engaging* phase, during which rapport is established, and the patient is encouraged to engage meaningfully with the counselor. During this phase, the counselor builds an interpersonal relationship with the patient (Miller and Rose, 2009). This is followed by a *Focusing* phase, where the counselor works to identify the key issues, their underlying causes, and the patient's background in order to define a clear focus for the conversation.

The third phase, *Evoking*, aims to foster the patient's intrinsic motivation for change. This is achieved by eliciting and reinforcing “change talk” dialogue acts, which are statements or behaviors indicating a desire to change the target behavior, as opposed to “sustain talk” dialogue acts, which reflect resistance or lack of readiness for change.

Finally, the *Planning* phase involves developing a concrete and actionable plan for behavior change, grounded in the patient's expressed motivation and goals.

Importantly, these phases are not strictly sequential. Counselors must ensure that certain goals, such as establishing sufficient engagement, clarifying key concerns, and generating adequate motivation, are met before progressing to the next phase. Furthermore, depending on the patient's evolving state of engagement and motivation, it may be necessary to revisit earlier phases. This non-linear and individualized progression underscores the need for flexibility.

For a virtual agent implementing MI, effectively navigating these phases is essential. It requires the ability to assess when to transition between phases, when to return to earlier stages, and how to adapt the interaction to meet each patient's specific needs and context.

### 3.2 Patients' profiles in MI

Patients participating in MI exhibit varying levels of readiness to change their behaviors. As proposed by Galland et al. (2025a), they can be classified into three profiles: *Open to Change, Resistant to Change*, and *Receptive*.

*Open to Change* individuals show a strong willingness to modify unhealthy behaviors. In contrast, *Resistant to Change* patients are generally reluctant to change, often preferring to maintain the status quo. *Receptive* patients, while initially showing low motivation, gradually develop a stronger desire to adopt healthier behaviors as the dialogue progresses.

These categories reflect differences in patient responses and in the corresponding strategies used by counselors, as discussed in Galland et al. (2025a). Being able to adapt the dialogue flow to these three patient profiles can significantly improve the effectiveness of the counselor's dialogue model (Galland et al., 2025a).

Therefore, a dialogue system for MI capable of addressing the unique challenges posed by these types of interactions could improve the effectiveness of the intervention. It should be able to navigate across the different dialogue phases and adapt its behavior to suit different user profiles.

## 4 Method

**Figure 2:**
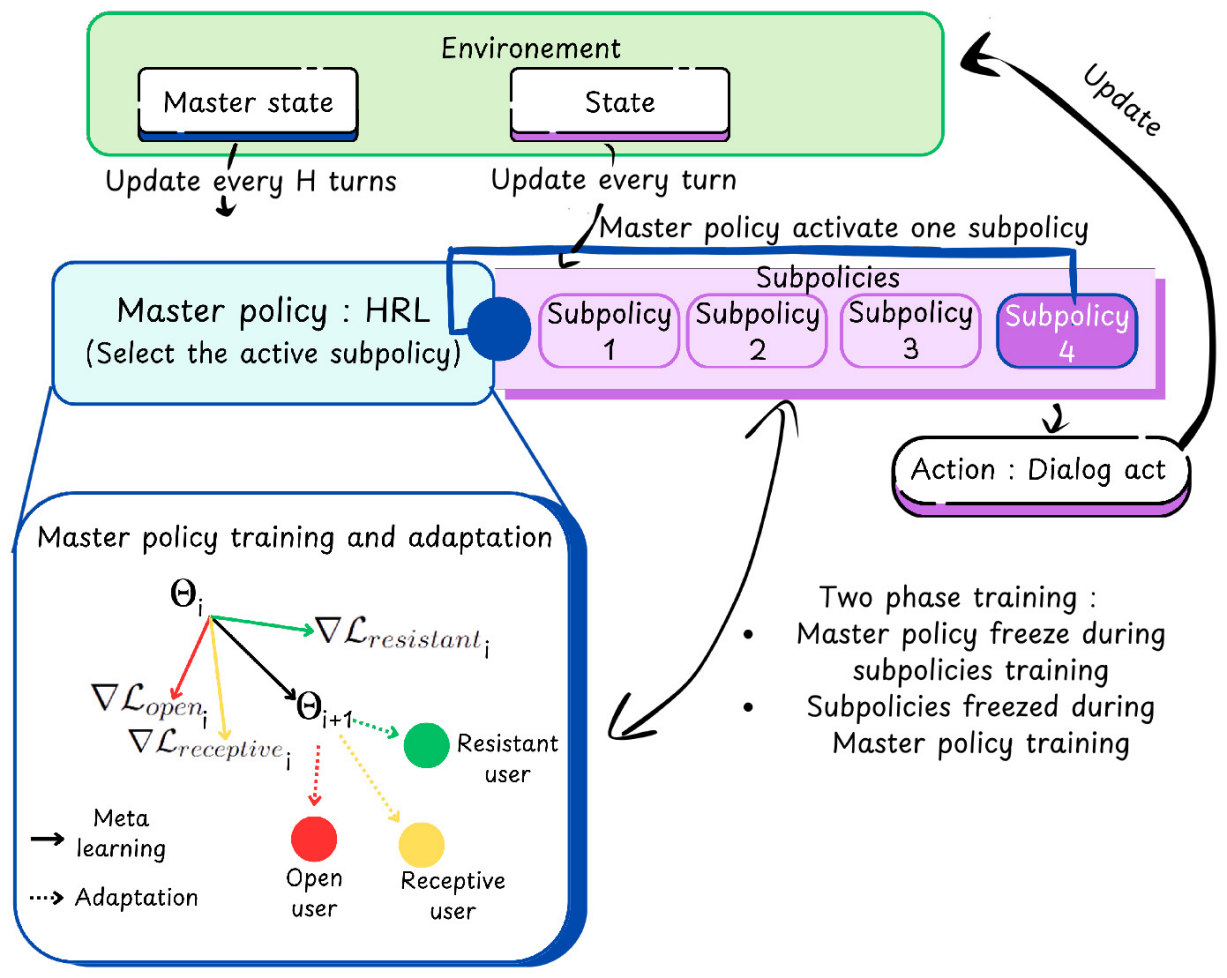
Hierarchical architecture of the dialogue manager.

This section outlines our methodology for developing a dialogue manager capable of navigating the distinct phases of dialogues (for the MI application: *Engaging, Focusing, Evoking*, and *Planning*, presented in Section 3.1), while adapting to different user profiles (for the MI application: *Open to Change, Receptive*, and *Resistant to Change*, presented in Section 3.2). The dialogue manager produces the next dialogue act (Schegloff, 1999) of the agent given the dialogue context. The complete architecture of the dialogue manager is illustrated in Figure 2 and explained in the following section.

### 4.1 Problem description

The objective of the proposed model is to predict the optimal dialogue act *a*_*t*_ at each time step *t*, given the dialogue context *c*_*t*_. This context includes the sequence of dialogue acts forming the dialogue history up to time *t*, as well as an embedding representing the last three dialogue turns. Additionally, the model considers the identified user profile and the current MI phase.

Each action corresponds to a dialogue act that represents the virtual agent's intent (e.g., Question, Greeting). This dialogue act is then used to condition an LLM to generate a natural language utterance that is both coherent with the context and aligned with the selected dialogue act. This approach to utterance generation from dialogue acts has been validated in Galland et al. (2024).

**Figure 3:**
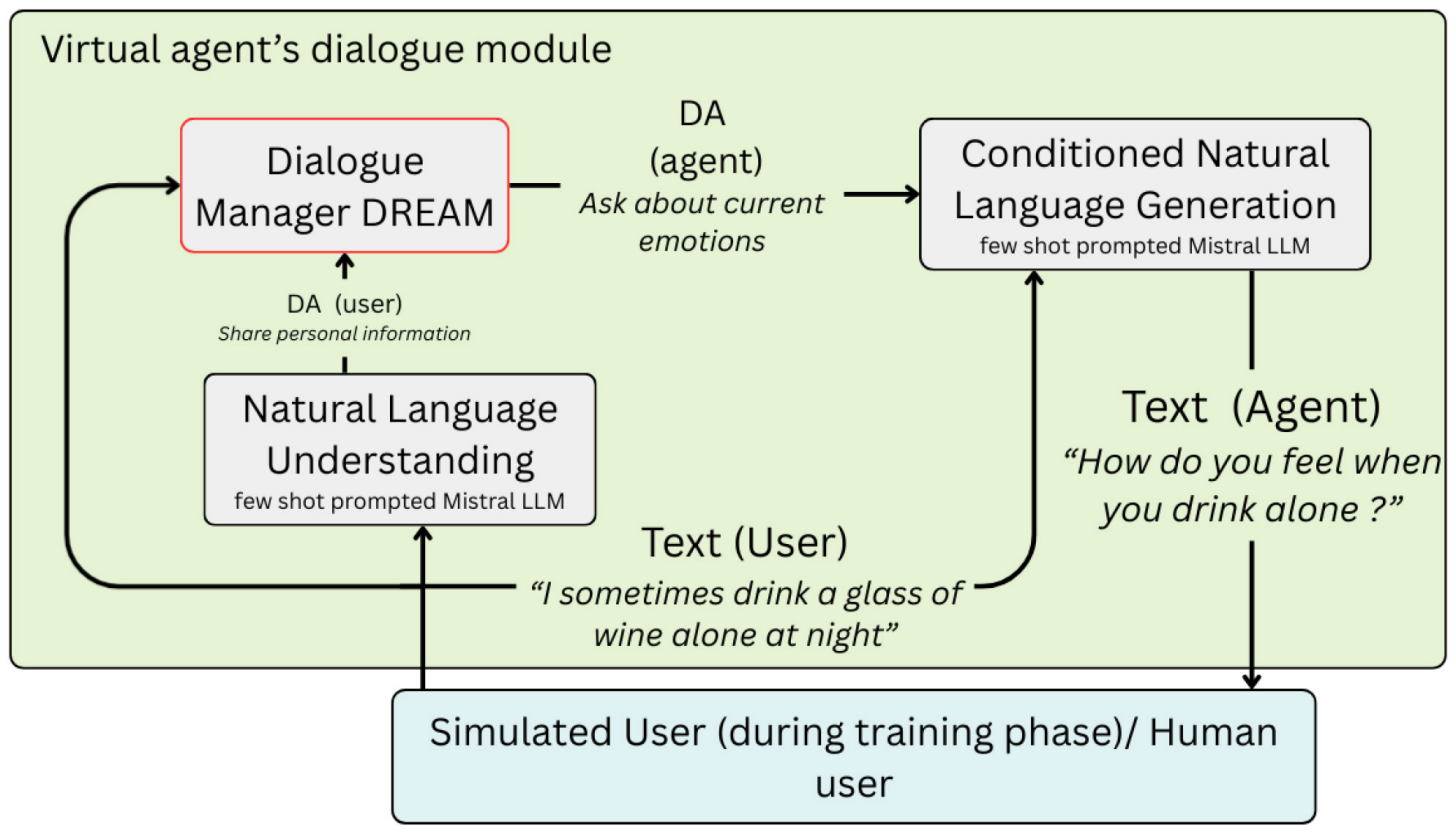
Overview of the system dialogue module architecture: integration of the dialogue manager with the Natural Language Understanding and Natural Language Generation modules, as well as with the simulated user/human user.

The generated utterance is processed by a simulated user (during the training phase) or a human user (during the evaluation phase), which produces the corresponding user response. This response is then interpreted by a Natural Language Understanding module, composed of an LLM with few-shot prompting and validated in Galland et al. (2025a), to extract the user's dialogue act. This act is passed to the dialogue manager, which uses it to predict the next optimal action. The overall system architecture is illustrated in Figure 3.

Since our application case is complex, relying on a rule-based dialogue manager would require a large number of handcrafted rules. Therefore, we opt for data-driven methods. Given our limitations in available data and the inherently interactive nature of the dialogue setting, we use reinforcement learning, as discussed in the related work section.

### 4.2 Hierarchical reinforcement learning for managing dialogue phases

The dialogue manager relies on a Hierarchical Reinforcement Learning (HRL) framework composed of a master policy and *N* sub-policies to structure the conversation into distinct phases of MI. HRL is particularly suited for this task because it decomposes prolonged, structured interactions into manageable sub-tasks. In this case, the sub-tasks represent the phases of the dialogue (for MI: *Engaging, Focusing, Evoking*, and *Planning*).

The goal is for each sub-policy to govern behavior within a specific dialogue phase, selecting appropriate dialogue acts in line with localized goals such as eliciting change talk for the *Planning* phase, for example, or affirming user reflections for the *Evoking* phase. The master policy oversees the higher-level strategy by deciding when to transition between phases, operating at a larger timescale than the sub-policies. Specifically, the master selects a phase for the next *H* turns based on a global context (master state, described in Section 5.2.2), which includes indicators of phase completion or user motivation trends (number of performed change talks). In contrast, the sub-policies' states (described in Section 5.2.2) represent local dialogue context, such as recent utterances, sentiment, or dialogue act history.

This structure balances short-term responsiveness with long-term planning. The fixed interval *H* reduces the frequency of the master policy decisions, simplifying the training process and ensuring that sub-policies have enough room to execute their strategies meaningfully. The value of *H* is empirically chosen to balance adaptability and coherence.

### 4.3 Meta-learning for user adaptation

To facilitate rapid adaptation to new users, the master policy is trained using meta-learning techniques, specifically the Model Agnostic Meta Learning (MAML) algorithm (Finn et al., 2017). MAML is designed to train models that can adapt quickly to new tasks using only a small amount of data. In our case, each user profile is treated as a separate task. Indeed, dialogues with different user profiles have common characteristics such as the different MI phases, the start and end of dialogue with Greeting and Closing, etc. However, there are also differences in the counselor's behavior depending on the patients' profiles. For instance, the counselor tends to use more empathic statements (i.e., engage more) with patients who are “Receptive” (Galland et al., 2025a). Due to these common characteristics and differences, the dialogue with each profile can be seen as a different task that needs to be learned. The goal of the training is then to find a master policy initialization that generalizes with a small amount of additional training across all the user profiles.

By leveraging MAML, the dialogue manager learns shared high-level strategies common to various user profiles while retaining the flexibility to fine-tune to individual users. After meta-training, the master policy is a well-informed starting point approximating an average strategy learned from prior users. It can rapidly adapt to a new user during deployment by performing a few gradient updates based on the first dialogue turns, enabling efficient personalization.

By isolating user-specific adaptation to the master policy, the system reduces adaptation complexity; only the high-level strategy layer needs fine-tuning to personalize the interaction, while sub-policies remain reusable. This modularity enhances generalization across user profiles and supports real-time, tailored transitions between MI phases, aligning with therapeutic goals and user needs.

### 4.4 Algorithm and training framework

In this subsection, we formally present our dialogue management algorithm and training procedure.

#### 4.4.1 Dialogue management algorithm

The model aims to predict the optimal dialogue act *a*_*t*_ at each time step *t* to maximize a reward function $R\left(s_{t},a_{t}\right)$, reflecting the interaction's therapeutic quality and effectiveness. The dialogue manager is structured hierarchically, with a master policy θ and a set of *N* sub-policies ψ_0_, …, ψ_*N*_.

The master policy operates over a discrete action space of size *N*. It selects which sub-policy to activate for the next fixed horizon of *H* turns. Each sub-policy ψ_*i*_ handles the generation of dialogue acts with an action space of size *N*_*da*_ = 13, corresponding to 13 predefined motivational interviewing dialogue acts of patients as defined in Section 5.2.2.

At each time step *t*, the algorithm proceeds as follows:

- If *t* mod *H* = 0

- the master policy observes the high-level master state $s_{t}^{master}$ and selects a new sub-policy: $A_{t}=\theta \left(s_{t}^{master}\right)$.

- Otherwise

- The master action remains unchanged: *A*_*t*_ = *A*_*t*−1_.

- The selected sub-policy ψ_*A*_*t*__ then takes the current sub-state *s*_*t*_ as input and generates a dialogue act: *a*_*t*_ = ψ_*A*_*t*__(*s*_*t*_).

This dialogue act *a*_*t*_ is executed by the agent, influencing the environment (i.e., a simulated user), which in turn produces a response and updates the states *s*_*t*+1_ and $s_{t+1}^{master}$. The system thus alternates between strategic planning (MI phase or master action selection) and fine-grained conversational control (dialogue act or action selection), enabling phase-aware and personalized dialogue progression. The complete training loop is described in Algorithm 1.

Algorithm 1 **Hierarchical Dialogue Management Algorithm.**

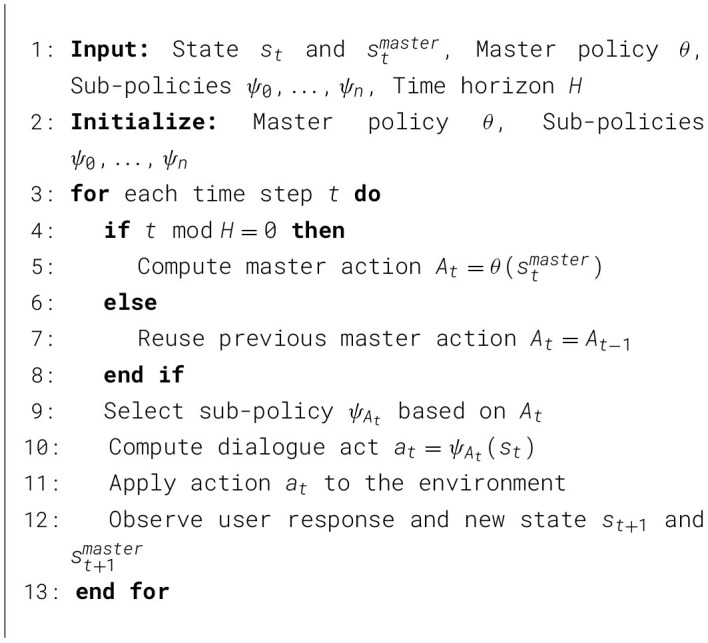

#### 4.4.2 Training framework

The training framework leverages a model-based RL approach, allowing efficient policy learning by simulating user interactions. In this context, the simulated environment enables the reuse of dialogue history across training epochs, enhancing data efficiency.

We adopt the Soft-Actor Critic (SAC) algorithm (Haarnoja et al., 2018), chosen for its off-policy nature and entropy-regularized objective, which supports stable learning and encourages diverse, exploratory behavior, crucial for training a generalizable and adaptable dialogue manager.

Each training epoch targets a specific user profile and begins with cloning the current master policy θ, creating a personalized variant θ_clone_. The optimization is performed in two distinct phases:

1. **Sub-policy optimization:** The cloned master policy θ_*clone*_ is kept fixed, while all sub-policies ψ_0_, …, ψ_*n*_ are trained using SAC to optimize phase-specific strategies for that profile.
2. **Master policy adaptation:** The trained sub-policies are frozen, and the cloned master policy θ_clone_ is fine-tuned using SAC. This phase focuses on adapting a high-level dialogue strategy to the profile-specific interaction dynamics.

After both optimization steps, the updated policies are evaluated, and the original master policy θ is updated using the MAML algorithm. This meta-learning step adjusts θ to improve its initialization, enabling rapid adaptation to unseen user profiles during deployment.

At inference, the master and subpolicies are fixed. Meta-learning is only used between dialogues to improve the master policy.

This modular and staged training design ensures that global (master policy) and local (sub-policies) strategies are learned effectively, while supporting scalable, real-time personalization for diverse user profiles. In this framework, the dialogue is fully generated automatically. The only fixed items are training parameters and the reward function. The whole training process is detailed in Algorithm 2.

Algorithm 2 **Training Process for Hierarchical Dialogue Manager.**

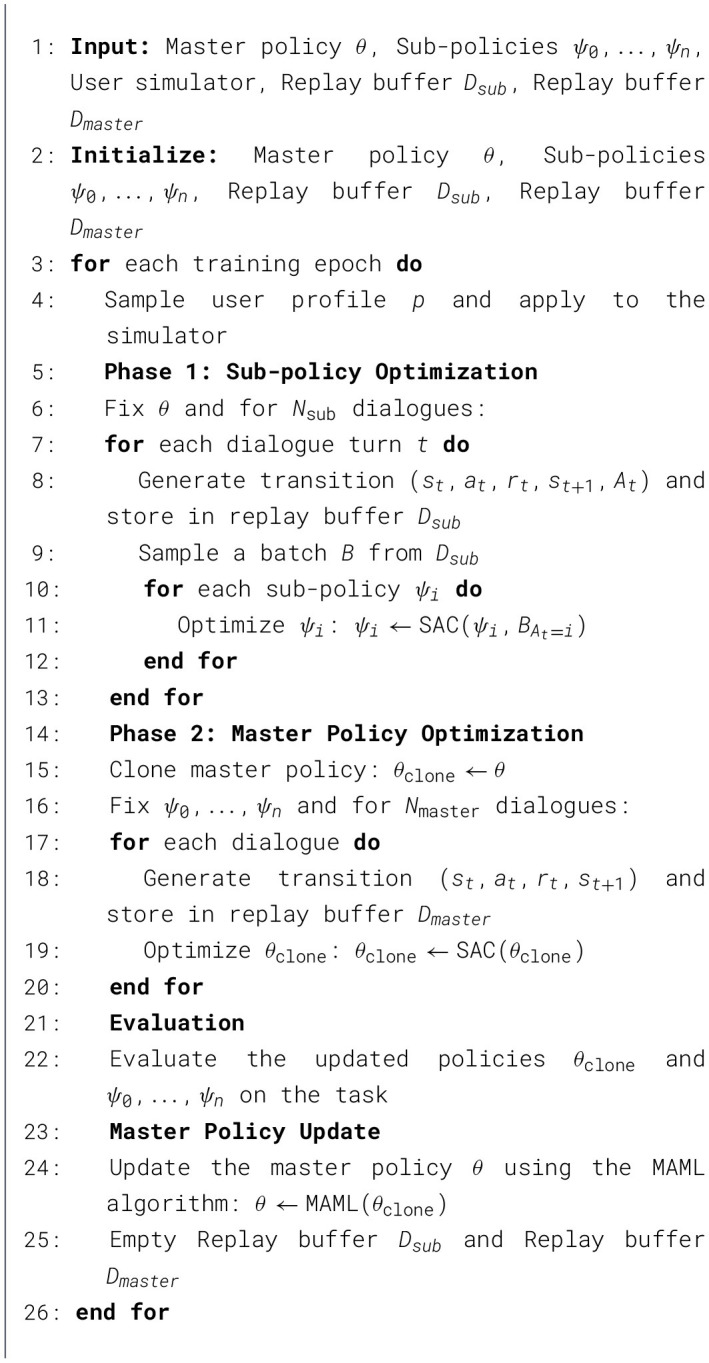

## 5 Evaluation environment

This section presents the evaluation setup used to assess our adaptive MI dialogue manager. We compare our model to a state-of-the-art LLM baseline and perform ablation studies to isolate the contribution of key architectural components. This evaluation is completed by an experimental study presented in Section 8.

### 5.1 Baseline

We use as a baseline the Nemo Instruct end-to-end LLM,^1^ prompted as described in Steenstra et al. (2024). The prompt incorporates MI principles and counseling strategies tailored to three behavior change topics: *Smoking, Alcohol*, and *Sedentary Lifestyle*. This baseline was validated with human participants in prior study (Steenstra et al., 2024) and is the best state-of-the-art baseline available at the time of the study. We aim to compare our approach of using external dialogue management with state-of-the-art end-to-end dialogue systems.

### 5.2 Simulated user

The evaluation environment includes a simulated user, described in Galland et al. (2024). This simulated user is composed of LLM (Mistral-7b-Instruct v2), prompted with information about MI, the current topic, and the profile of the user. This simulated user has been tested and validated by objective evaluation and a user study. The results show that the behaviors produced by the simulated user align with those of a human (Galland et al., 2024). In an initial step, we utilize a simulated user for training and initial evaluation; interacting with a human user can be more complex. Thus, we employ a simulated user as a proxy. We confirm the validity of this approach through an experimental study in Section 8. The dialogue manager interacts with the simulated user during the training to infer the impact of each possible action on the dialogue. At each new dialogue, the simulated user is randomly initialized with a specific behavior change topic *T*∈{Smoking, Alcohol, Sedentary Lifestyle} and a user profile *P*∈{Receptive, Resistant to Change, Open to Change}. A full dialogue is generated between the agent and the simulated user for each test session, with a maximum of 20 agent dialogue turns.

#### 5.2.1 Action space

For the application case of MI, the agent (or virtual counselor) operates in a discrete action space consisting of 13 possible dialogue acts, categorized into task-oriented and socially oriented dialogue acts according to the taxonomy introduced in Galland et al. (2025a). Task-oriented dialogue acts include *Asking for Consent or Validation, Providing Medical Education and Guidance, Planning with the Patient, Giving a Solution, Asking about Current Emotions, Inviting a Shift in Outlook, Asking for Information*, and *Reflection*. Socially oriented dialogue acts include *Empathic Reactions, Acknowledging Progress and Encouragement, Backchanneling, Greeting or Closing, and Normalizing Experiences while Providing Reassurance*.

#### 5.2.2 State space

The agent's state space includes the most recent agent and user dialogue acts. As defined in Galland et al. (2025a), the simulated user (or patient) can use 9 different dialogue acts, such as *Changing Unhealthy Behavior, Sustaining Unhealthy Behavior, Sharing Negative/Positive Feelings or Emotions, Sharing Personal Information, Realization or Understanding, Greeting or Closing, Backchanneling*, and *Asking for Medical Information*. In addition, the state incorporates the current turn number and an encoded representation of the dialogue context, which includes the last three utterances(the number of utterances is chosen to give enough context while keeping an acceptable input size). This context is encoded using sentence embeddings from the Nomic embed-text model,^2^ which capture semantic similarity and ensure continuity in the agent's responses. So, the sub policies state space is:

$S=\left\{DA_{agent}^{t-1},DA_{user}^{t-1},t,Embedding\left(\text{last 3 turns}\right)\right\}$

#### 5.2.3 Master state space

The master policy's state space includes high-level approximations of the simulated user's progression through the MI process: Context knowledge, Engagement, and Evocation. These are estimated as follows: *Context knowledge* is approximated by counting occurrences of the *Sharing Personal Information* act; *Engagement* by the number of times the user expresses *Positive/Negative Feelings*; and *Evocation* by the frequency of the *Realization or New Perspective* act. These features were selected to reflect MI phase progression, enabling the master policy to monitor therapeutic engagement and adapt the interaction accordingly. In summary, the master policy state space is:

$S_{master}=\left\{N_{Sharedinformation}^{U},N_{Expressedfeeling}^{U},N_{Gotnewperspective}^{U}\right\}$

#### 5.2.4 Reward function

The reward function is designed to estimate the therapeutic success based on the user's dialogue acts. Prior research highlights the impact of user language: *Sustain talk* correlates with poor outcomes (Magill et al., 2014), while *change talk* predicts positive behavior change (Magill et al., 2018). Accordingly, a reward of +5 is assigned for *Changing Unhealthy Behavior*, and a penalty of −5 for *Sustaining Unhealthy Behavior*. Additional structured rewards guide the dialogue through MI phases:

- When *Engaging* is needed (when the user expressed less than 2 emotions), Sharing positive/Negative feelings or emotions yields +50.
- When *Focusing* is needed (when the user is engaged and shares less than 2 pieces of information), Sharing personal information yields +100.
- When *Evoking* is needed (when the user is engaged and focused and shared less than 2 reflections), A Reflection from the user earns +150.
- When *Planning* is needed (when all the above conditions are met), the user planning change yields +200.

**Figure 4:**
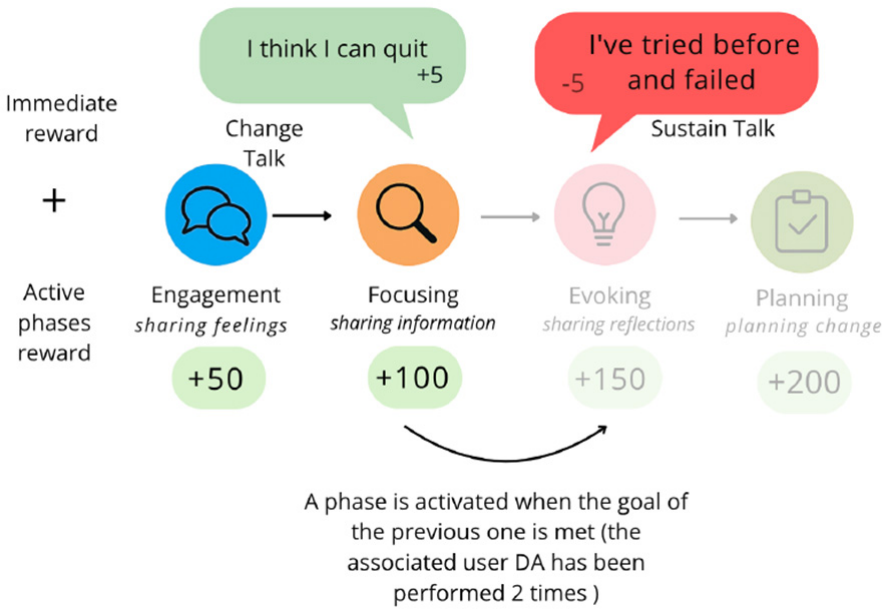
Illustration of the reward function with an immediate reward for *Change Talk* and *Sustain talk* and a reward associated with active phases.

Thus, the reward is composed of immediate rewards or penalties based on the detection of *Change Talk* or *Sustain Talk*, and rewards associated with phase transitions. A phase is considered complete, and the subsequent phase is activated, once the target user dialogue act has been performed two times. This process is detailed in Figure 4.

These reward values are empirically tuned to strike a balance between learning stability and goal alignment. The value of the phase-related reward increases with the progression of the dialogue, as later subgoals are more challenging to reach than early subgoals. For the same reason, phase-related rewards are more valuable than direct change talk-related rewards.

#### 5.2.5 Episode termination

An episode concludes either when the agent performs a closing action or after 20 agent dialogue turns, beyond which the interaction would be considered excessively long.

### 5.3 Hyperparameters

The model is trained for 41 epochs, with 455 conversations per epoch. Of these, 300 are used to train the master policy, 150 to train the sub-policies, and 5 for evaluation. This split was chosen empirically to balance global and local policy learning. To accelerate training, five conversations are run in parallel. The model includes six sub-policies, with the master policy invoked every three turns (*H* = 3). We choose to select a master policy every three dialogue turns to balance the training horizon and prevent the repetition of the same policy for too long. Each user's profile is sampled randomly at the start of each epoch.

Sub-policies are trained using a learning rate of 10^−4^ and a batch size of 1000. The master policy uses a learning rate of 10^−3^ and the same batch size. The MAML algorithm operates with a learning rate of 4 × 10^−4^. All networks have two linear layers with Leaky ReLU activations and a hidden size of 32. Training is conducted over 16 hours on a 42GB GPU.

## 6 Objective evaluation on simulated evaluation environment

**Figure 5:**
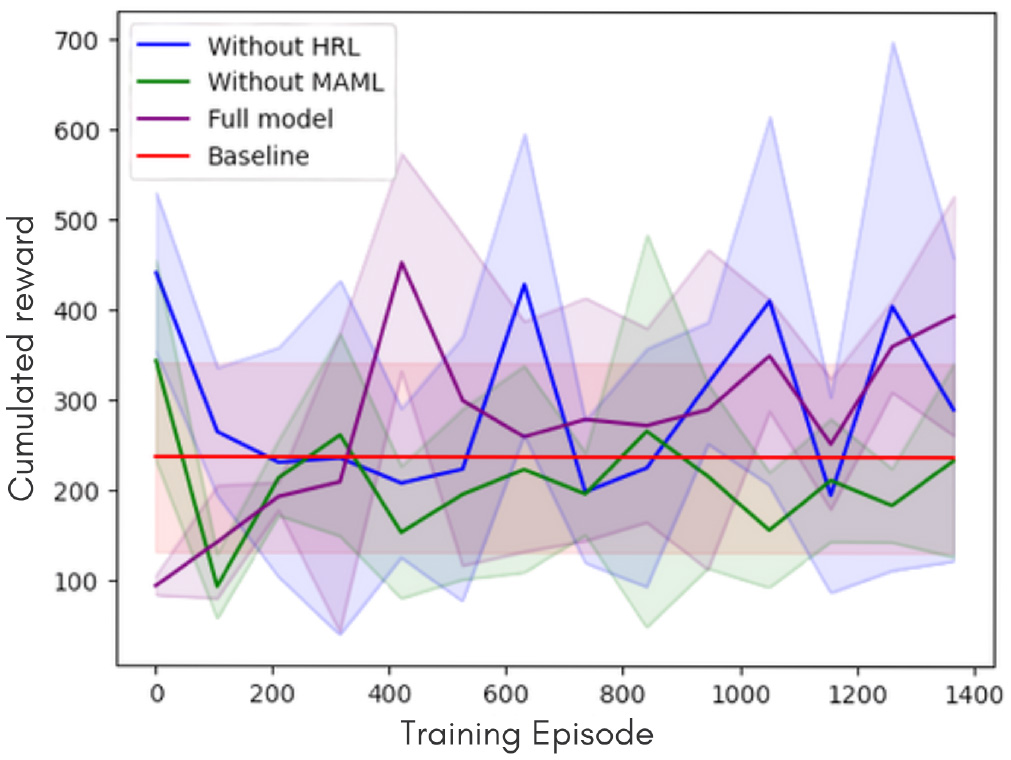
Evolution of the reward function during training; the baseline reward is the mean reward value of 100 dialogues realized with the baseline LLM. It does not evolve with the training epoch, as the baseline does not have any training.

In this section, we present our results and the results of ablation studies. Figure 5 illustrates the mean reward evolution across all three user profiles, *Open to Change, Resistant to Change*, and *Receptive*, throughout the training process. At each evaluation epoch, five conversations are conducted with each user profile. Additionally, Table 1 presents the final experimental results. Our model's reward performance surpasses the baseline, demonstrating that conditioning an LLM with our dialogue manager improves the fulfillment of MI goals in the dialogue compared to using a plain end-to-end LLM. We observe high variability in reward values throughout the training process. This fluctuation is primarily due to the inherent stochasticity of dialogue environments. Indeed, the same agent action in a given state can lead to different user responses. Such variability introduces additional challenges to the training process, making it more difficult to achieve consistent improvement in this task. Moreover, due to computational constraints, only five dialogues are conducted at each evaluation epoch. Such a low number cannot represent the variability of potential dialogues, thus further contributing to the observed variance. Despite this variability, an upward trend in performance is observed throughout training. The RL model consistently outperforms the baseline in terms of average reward, demonstrating its effectiveness in promoting desirable dialogue behaviors.

**Table 1:** Experiment results with mean rewards (. = difference with the baseline *p* < .1).

Experiment	Mean reward (±SD)
Baseline	235± 106
Without MAML	233 ± 106
Without HRL	290 ± 169
Full model	**394** **±132** (.)

The higher value is bolded.

### 6.1 Ablation studies

We conducted two ablation studies to evaluate the individual contributions of our design choices. In the first study, we use the same training framework without the use of MAML to isolate the impact of meta-learning. In the second study, we removed the HRL framework entirely and trained the agent using only the SAC algorithm (see Figure 5 and Table 1). These comparisons allow us to decouple the benefits of the hierarchical structure from those of the meta-learning approach.

#### 6.1.1 Effect of MAML

The inclusion of MAML improves the accumulated reward (see Table 1), suggesting that it enhances the learning of the master policy by explicitly accounting for variations across user profiles. Standard training can be biased by the sequence in which different user profiles are encountered. However, MAML mitigates this by guiding the master policy toward an initialization that enables rapid adaptation to diverse user profiles.

#### 6.1.2 Effect of HRL

The experimental results further support the effectiveness of HRL. Training with only the SAC algorithm leads to a lower accumulated reward, likely because the phase-based structure of MI dialogues, with a set of subgoals to fulfill, is more challenging to capture without hierarchical modeling.

## 7 Interpretation

This section analyses the generated dialogues and examines how our design choices influence them. We investigate the different MI phases to determine whether they emerge as expected and assess the impact of HRL. Additionally, we explore variations across user profiles and evaluate the effect of MAML on the generated dialogues.

### 7.1 Differences between phases

**Figure 6:**
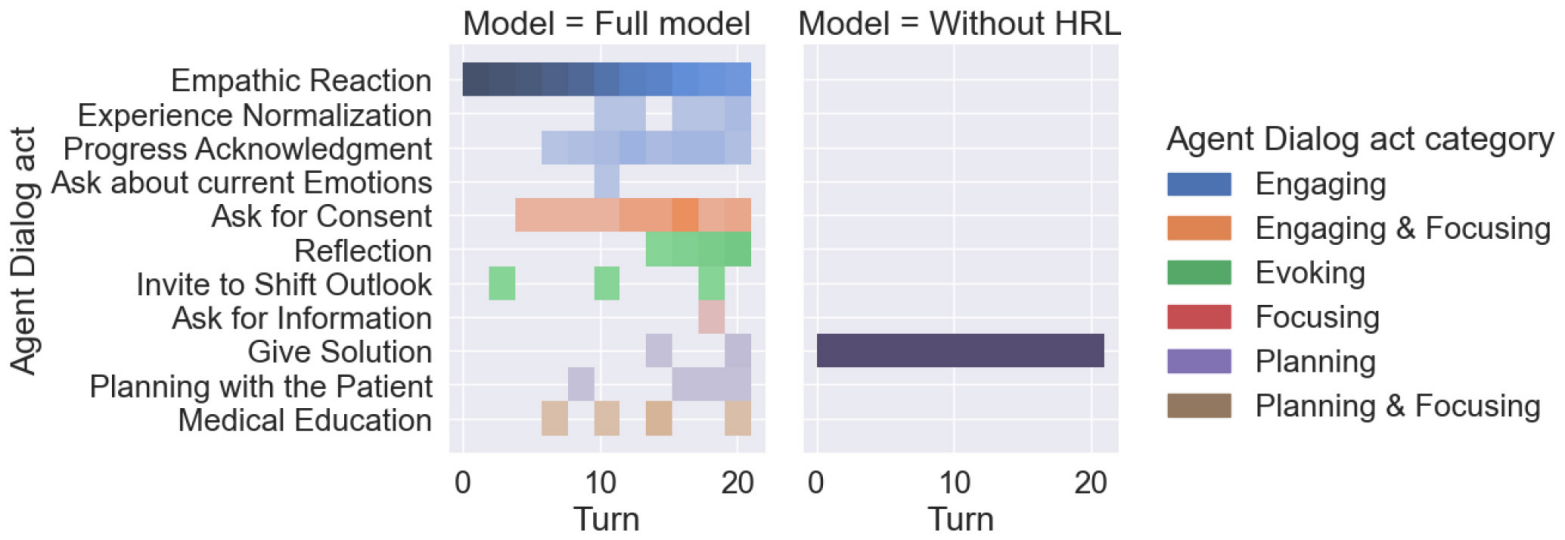
Dialogue act distribution over time, highlighting different dialogue phases for the full model. The intensity of the color is proportional to the use of the corresponding dialogue act in the turn.

We examine the distribution of dialogue acts across different dialogue turns to analyze the MI phases. Figure 6 shows that the full model uses engagement-related dialogue throughout the whole dialogue and uses *Focusing*-related actions in the last three quarters of the interaction. It also uses *Evoking* and planning-related actions toward the end of the interaction. This is aligned with MI principles as dialogue acts associated with the *Engaging* phase, such as asking about feelings or emotions, should be more prevalent at the beginning of the conversation. In contrast, those related to the *Planning* phase, such as providing solutions or promoting behavior change, should appear more frequently toward the end of the interaction (Miller and Rollnick, 2012). While engagement should occur throughout the entire dialogue, the later stages should focus more on planning; once engagement is established, it requires less focus to maintain. The phases are interwoven rather than strictly sequential. However, the HRL ablation only uses a single action: “Give solution”. This strategy may provide immediate rewards with the simulated users, as it gives immediate solutions, but does not adhere to the MI principle. Indeed, MI is collaborative, and the user should come up with solutions on their own. This shows that the full model with HRL is more diverse and MI-compliant.

### 7.2 Differences between user profiles

**Figure 7:**
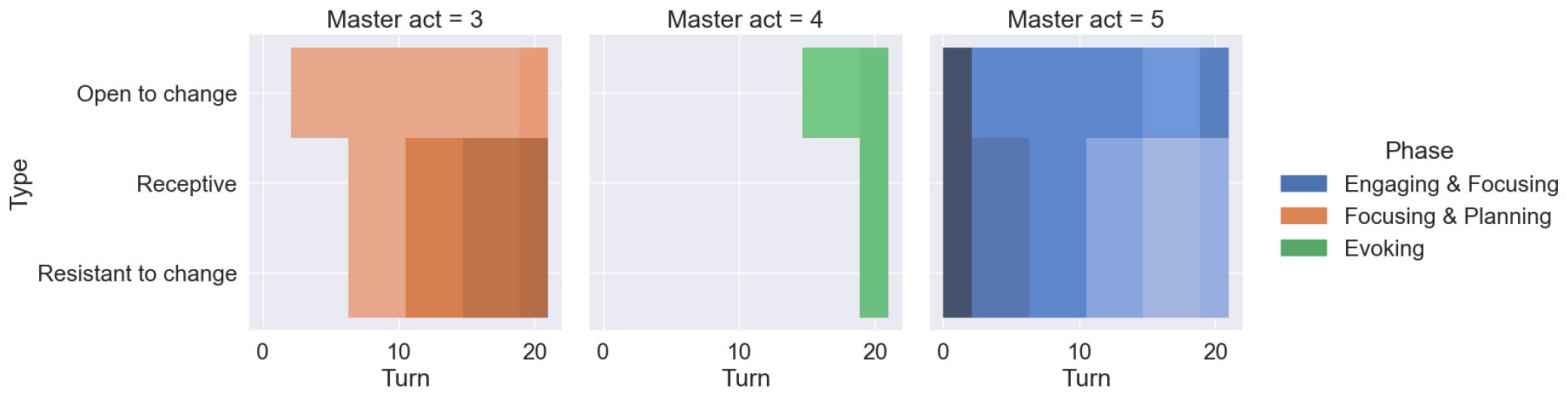
Distribution of phase activation over time across different user profiles. The intensity of the color is proportional to the use of the phase in the turn.

In this section, we examine the impact of meta-learning on training the master policy. For the model trained without meta-learning, we observe a collapse of the master policy to a single dominant action across interactions. Figure 7 illustrates the distribution of activation of master actions over time for different user profiles for the full model. This highlights the difficulty of learning a generalized policy that performs well across diverse user profiles without explicit mechanisms for adaptation. In contrast, meta-learning allows the master policy to maintain variability and adaptability in its actions. In Figure 7, we associate each master action with MI phases based on the predominant dialogue acts typically observed in those phases and master actions, providing interpretability into the policy's learned structure. The models effectively differentiate between distinct phases, initially *Engaging* in *Engaging*/*Focusing* phases (Master action 5) before transitioning into *Focusing*/*Planning* phases (Master action 3) and *Evoking* phase (Master action 4). The *Engaging* phase is shorter, with the frequency of *Engaging* related dialogue acts quickly decreasing, for *Open to Change* users, as they do with a human counselor, aligning with prior findings (Galland et al., 2025a). Indeed, *Open to Change* users require less rapport-building with the counselor, as they tend to initiate the conversation themselves. This qualitative analysis highlights the benefits of incorporating meta-learning, as it enhances the model's ability to structure MI phases effectively and provide a more personalized dialogue experience. Virtual agents interacting with *Open to Change* patients tend to use fewer dialogue acts oriented toward *Focusing* and *Planning*, perhaps because these patients are more capable of independently planning.

## 8 Subjective evaluation: experimental study

The previous sections presented our dialogue manager DREAM. The objective evaluation demonstrated that DREAM is able to navigate through dialogue phases and adapt to different types of simulated users. In this section, we present a subjective evaluation of the DREAM dialogue manager to determine whether these results are transferable to interaction with human users. The evaluation is conducted by integrating the manager into the Greta 2.0 platform, an embodied virtual agent environment described in Section 8.1, and performing a human-agent study, detailed in Section 8.4.

### 8.1 Integration with the Greta 2.0 platform

The evaluation was performed within the Greta 2.0 platform (Saga et al., 2025), an open-source framework for embodied virtual agents. The code of Greta and DREAM is available at https://github.com/isir/greta. Embodiment has been shown to enhance the perception of an agent for therapeutic support (Lim et al., 2025). Greta provides modular components for managing turn-taking and verbal and non-verbal behavior. We integrated the DREAM dialogue manager, along with Natural Language Generation (NLG) and Natural Language Understanding (NLU) modules powered by a Mistral large 2.1 version of November 2024.

#### 8.1.1 Natural Language Understanding (NLU)

The NLU module uses few-shot prompting with definitions and examples for each dialogue act. This method has been validated in Galland et al. (2025a) with a macro F1 score of 0.7. The prompts used for NLU are detailed in Appendix 1.

#### 8.1.2 Natural Language Generation (NLG)

The NLG module uses an LLM prompted with MI principles and domain-specific information (e.g., smoking, alcohol use, sedentary lifestyle). The prompt associated with the Baseline is based on Olafsson et al. (2020). For the DREAM condition, the prompt is extended with a few-shot example to guide generation toward a specific dialogue act. The dialogue context is also included. This approach was validated in Galland et al. (2024). Full prompts are available in Appendix 2, and Table 2 summarizes the template structure.

**Table 2:** Prompt templates for the two conditions of the study.

Condition	Prompt template
Base	Your name is Dr. Anderson. You will act as a skilled therapist conducting a Motivational Interviewing (MI) session focused on smoking cessation. The goal is to help the client identify a tangible step to reduce smoking within the next week. The client's primary care doctor referred them to you for help with their [Theme] habit. Start the conversation with the client with some initial rapport building, such as asking, How are you doing today? (e.g., develop mutual trust, friendship, and affinity with the client) before smoothly transitioning to asking about their smoking. Keep the session under 15 minutes and each response under 150 characters long. In addition, once you want to end the conversation, add END_CONVO to your final response. You are also knowledgeable about [Theme], given the Knowledge Base - [Theme] in the context section below. When needed, use this knowledge about [Theme] to correct any client's misconceptions or provide personalized suggestions. Use the MI principles and techniques described in the Knowledge Base - Motivational Interviewing (MI) context section below. However, these MI principles and techniques are only for you to use to help the user. These principles and techniques, as well as motivational interviewing, should NEVER be mentioned to the user.
Baseline	Base prompt + {MI knowledge Base} + {Subject knowledge Base} + {Patient type definition} < < < Context: {context} Generate the therapist's next utterance. >>> Therapist's utterance:
Dialogue act conditioned	Base prompt+ {MI knowledge Base} + {Subject knowledge Base} + {Utterance examples with corresponding dialogue acts} < < < Context: {context} Generate the patient's next utterance with the intent: {intent} >>> Therapist's utterance:

Prompt templates for the two conditions of the study.

#### 8.1.3 MODIFF-8 facial expression model

The MODIFF-8 model (Younsi et al., 2025) generates facial expressions that are suitable for MI interactions. It is built on a diffusion model that has been trained using MI interaction videos. This model produces the facial expressions of the agent based on the expressions of the user in real-time. Additionally, it adjusts the agent's facial expressions frame-by-frame to ensure they are both expressive and synchronized with the user's expressions.

### 8.2 Text to speech

The text is turned into audio by Greta 2.0's Text-to-Speech module. This module uses Cereproc (Aylett and Pidcock, 2007) to turn text into audio with automatic lip synchronization.

### 8.3 Automatic speech recognition

The user's audio is turned into text by Greta 2.0's Automatic Speech recognition module. It uses the multilingual API of Deepgram to transcribe the audio into text.

### 8.4 Human-agent evaluation

In this section, we present an experimental evaluation conducted with the Greta 2.0 platform.

### 8.5 Protocol

During the study, participants begin by selecting a topic for discussion, which can be reducing smoking, reducing alcohol consumption, or increasing physical exercise. They then complete a questionnaire (described in Section 8.6) to assess their profile. Following this, participants interact with the Greta 2.0 agent, as detailed in Section 8.1. After the interaction, they fill out additional questionnaires to evaluate their perceptions of the interaction and their motivation to change behavior, as described in Section 8.6.

We conduct a between-subjects human-agent evaluation to assess the impact of our DREAM dialogue manager in two experimental conditions:

- Baseline condition: Dialogue is generated by a plain LLM prompted with MI-related information. This setup is based on the validated method described in Olafsson et al. (2020).
- DREAM condition: The DREAM dialogue manager selects the next dialogue act. A Mistral LLM then generates the corresponding utterance, conditioned on this selected act. We use three versions of the DREAM dialogue manager, which are derived from the same original policy and fine-tuned with users of each profile (*Open to Change*, *Receptive*, and *Resistant to Change*) for 100 conversations. The participants interact with the dialogue manager, which is fine-tuned for their identified profile.

### 8.6 Measures

We employ three subjective measures to evaluate the effectiveness of the dialogue manager. These measures are collected through validated questionnaires.

#### 8.6.1 Decisional balance scale

To assess participants' motivation before and after the interaction, we use the short version of the Decisional Balance Scale (DBS), adapted for smoking (Velicer et al., 1985), alcohol consumption (Carey et al., 2001), or exercise (Eeckhout et al., 2013), depending on the topic selected by the participant. The DBS is a 10-item self-reported questionnaire using a 5-point Likert scale (1 = Not important, 5 = Extremely important). It consists of five positive and five negative items. The DBS score is calculated by subtracting the average score of the negative items from the average score of the positive items. The items are visible in Table 3.

**Table 3:** DBS Survey statements, their polarity, mean scores, and 95% confidence interval for baseline and model conditions after intervention.

Survey statement	Polarity	Mean baseline score	Mean model score
Sedentary lifestyle			
I would have more energy for my family and friends if I exercised regularly.	Positive	3.04 [2.76; 3.33]	3.42 [3.16; 3.67]
At the end of the day, I would be too exhausted to exercise.	Negative	-3.04 [-2.77; -3.31]	-3.125 [-3.34; -2.91]
I would feel more confident if I exercised regularly.	Positive	3.70 [3.42; 3.97]	4.29 [4.13; 4.46]
I would sleep more deeply if I exercised regularly.	Positive	4.17 [3.96; 4.39]	3.92 [3.74; 4.09]
I would find it easier to complete daily physical tasks if I exercised regularly.	Positive	3.52 [3.28; 3.76]	3.75 [3.51; 3.99]
I would feel less stressed if I exercised regularly.	Positive	3.83 [3.56; 4.09]	4.08 [3.87; 4.30]
Regular physical activity would take up too much time.	Negative	-2.91[-3.16;-2.67]	-2.88[-3.10;-2.65]
I would have less time for my family and friends if I exercised regularly.	Negative	-2.48 [-2.69; -2.26]	-2.42 [-2.64; -2.19]
I would be too tired to do my daily work after exercising.	Negative	-2.96 [-3.24; -2.67]	-2.92 [-3.15; -2.68]
I would have difficulty finding a physical activity that I enjoy, and that is not dependent on weather conditions.	Negative	-2.56 [-2.79; -2.33]	-2.63 [-2.86; -2.39]
Total Score		4.30 [3.11; 5.49]	5.50 [4.28; 6.72]
Drinking			
My alcohol consumption causes problems with others.	Negative	-2.25 [-3.20; -1.30]	
Drinking helps me cope with problems.	Positive	1.75 [1.00; 2.50]	
Drinking allows me to have fun and socialize.	Positive	3.50 [2.85; 4.15]	
Alcohol consumption disrupts my functioning at home and/or work.	Negative	-3.00 [-3.91; -2.09]	
Some of my loved ones are disappointed by my alcohol consumption.	Negative	-2.25 [-3.20; -1.30]	
Drinking helps me relax and express myself.	Positive	3.50 [2.63; 4.36]	
I feel like I get into trouble when I drink.	Negative	-2.50 [-3.46; -1.54]	
I could accidentally hurt someone because of my alcohol consumption.	Negative	-2.75 [-3.78; -1.72]	
I feel more confident when I drink.	Positive	3.75 [2.80; 4.70 ]	
Without alcohol, my life would be dull and boring.	Positive	3.75 [2.80; 4.69]	
Total Score		3.25 [-4.09; 10.59]	
Smoking			
Smoking cigarettes relieves tension.	Positive		
I am embarrassed about having to smoke.	Negative		
Smoking helps me concentrate and work better.	Positive		
My cigarette consumption bothers others.	Negative		
I am relaxed and therefore more pleasant when I smoke.	Positive		
People think I am foolish for ignoring cigarette warnings.	Negative		
Smoking cigarettes is dangerous for my health.	Negative		
If I try to quit smoking, I will be irritable and difficult to live with.	Positive		
Smoking cigarettes is enjoyable.	Positive		
My smoking affects the health of others.	Negative		

We only report the values for exercise as there are not enough participants in the other themes.

#### 8.6.2 Rapport

Rapport is measured using a subset of items from the Rapport Scale 2 (Wang and Gratch, 2009), a 6-item self-reported questionnaire employing a 5-point Likert scale (1 = Totally disagree, 5 = Totally agree). This measure assesses the participant's sense of connection and mutual understanding with the agent. The items are visible in Table 4.

**Table 4:** Rapport Scale Survey statements, and mean scores and 95% confidence interval for baseline and model conditions.

Survey statement	Mean baseline score	Mean model score
I felt I was able to engage the listener with my story.	3.08 [2.64; 3.33]	3.44 [3.25; 3.64]
I felt that the listener was interested in what I was saying.	3.50 [3.20; 3.71]	3.96 [3.79; 4.14]
I felt I had a connection with the listener	2.15 [1.90; 2.35]	2.56 [2.35; 2.76]
I think that the listener and I understood each other.	3.65 [3.30; 3.85]	3.67 [3.49; 3.84]
I was able to say everything that I wanted to say.	3.04 [2.65; 3.32]	3.04 [2.79; 3.28]
The listener was warm and caring.	2.85 [2.58; 3.08]	3.89 [3.73; 4.04]
Total Score	3.04 [2.77; 3.21]	3.43 [3.29; 3.56]

#### 8.6.3 Client evaluation of MI

To evaluate the perceived quality of MI, we use the Client Evaluation of Motivational Interviewing (CEMI) scale (Madson et al., 2015). The CEMI consists of 16 self-reported items rated on a 4-point Likert scale (1 = Never to 4 = A Great Deal), with 8 positive and 8 negative items. Following the standards set in Olafsson et al. (2020), we use a therapeutic threshold at the midpoint of the scale (CEMI = 2.5); dialogues rated above this threshold are considered to respect MI principles. The items are visible in Table 5. These three measures allow us to assess both the agent's effectiveness in delivering MI and the extent to which rapport is established during the interaction.

**Table 5:** CEMI Survey statements, their polarity, and mean scores and 95% confidence interval for baseline and model conditions.

Survey statement	Polarity	Mean baseline score	Mean model score
Focused on your weakness.	Negative	-1.62 [-1.81; -1.43]	-2.19 [-2.37; -2.00]
Help you talk about changing your behavior.	Positive	3.17 [3.01; 3.33]	3.07 [2.90; 3.24]
Act as a partner in your behavior change.	Positive	2.83 [2.66; 3.00]	2.85 [2.67; 3.03]
Help you discuss your need to change your behavior.	Positive	2.79 [2.59; 2.99]	3.00 [2.82; 3.18]
Make you feel distrustful of him/her.	Negative	-1.72 [-1.92; -1.53]	-1.66 [-1.84; -1.50]
Help you examine the pros and cons of changing your behavior.	Positive	2.31 [2.08; 2.54]	2.56 [2.36; 2.75]
Help you to feel hopeful about changing your behavior.	Positive	2.90 [2.72; 3.07]	3.26 [3.10; 3.42]
Argue with you to change your behavior.	Negative	-2.45 [-2.62; -2.27]	-2.78 [-2.99; -2.56]
Change the topic when you become upset about changing your behavior.	Negative	-1.28 [-1.39; -1.17]	-1.41 [-1.56; -1.26]
Push you forward when you become unwilling to talk about an issue further.	Negative	-2.03 [-2.20; -1.87]	-2.63 [-2.84; -2.42]
Act as an authority on your life.	Negative	-1.52 [-1.67; -1.37]	-1.41 [-1.53; -1.28]
Tell you what to do.	Negative	-2.79 [-2.99; -2.60]	-3.07 [-3.27; -2.87]
Argue with you about needing to be 100% ready to change your behavior.	Negative	-1.10 [-1.18; -1.03]	-1.15 [-1.24; -1.06]
Show you that she/he believes in your ability to change your behavior.	Positive	3.07 [2.85; 3.28]	3.44 [3.29; 3.60]
Help you feel confident in your ability to change your behavior.	Positive	2.72 [2.54; 2.90]	3.07 [2.91; 3.23]
Help you recognize the need to change your behavior.	Positive	2.24 [2.02; 2.46]	2.96 [2.80; 3.13]
Total Score		1.25 [1.08; 1.43]	1.32 [1.17; 1.48]

### 8.7 Recruitment

The protocol of this study has been validated by the INSEAD Institutional Review Board. Participants were recruited through the INSEAD-Sorbonne University Lab and compensated at standard rates ($13 per hour). They were healthy individuals drawn from the general population. A total of 62 participants were recruited; six were excluded due to technical issues during the intervention. The final sample consisted of 56 valid participants (55% female, 39% male, 4% non-binary, 2% preferred not to disclose), aged between 18 and 45 years, all of whom reported French as their primary language.

Of these, 27 participants interacted with the DREAM condition, and 29 with the Baseline condition. Participants were evenly distributed across conditions to ensure balance in gender and profile. A Chi-square test revealed no significant association between gender and condition. χ^2^(2, *N* = 56) = 3.15, *p* = 0.370, indicating independence between the two variables.

### 8.8 Participant profile identification

Based on the initial DBS score (pre-interaction), each participant is classified into one of three profiles: *Resistant to Change*, *Receptive*, or *Open to Change* (see Section 3.2). These profiles relate to stages of behavior change, which describe a progression individuals follow when attempting to modify behavior. The stages include: precontemplation, contemplation, preparation, action, maintenance, and sometimes termination, reflecting a continuum from no intention to change to sustained behavioral change (Prochaska and DiClemente, 1983).

As noted in Eeckhout et al. (2013), DBS scores correlate with these stages. Specifically, participants in the Precontemplation stage (DBS = 0.46 ±1.03), characterized by low awareness or knowledge of the issue, align with the *Receptive* profile. Those in the Contemplation stage (DBS = 1.27 ± 1.02), who recognize a problem and consider change, align with the *Open to Change* profile. In that study, no participants were identified as completely unwilling to change, corresponding to the *Resistant to Change* profile. Based on these findings, we define the profiles as follows:

- *Resistant to Change* if the initial DBS score is negative.
- *Receptive* if the score is between 0 and 2.
- *Open to Change* if the score is above 2.

### 8.9 Results

In this section, we present the results of our study along with the associated statistical analyses. The full questionnaire and corresponding results are presented in Table 3 for DBS, Table 4 for the Rapport scale, and Table 5 for CEMI [This study was originally presented in Galland et al. (2025b).

#### 8.9.1 Profile and theme repartition

Most participants chose to discuss physical exercise (84%), while 5% discussed smoking, and another 11% discussed alcohol consumption.

The distribution of detected users' profiles was 71% “Open to change”, 13% “Receptive”, and 16% “Resistant to change”. Users' profiles were evenly distributed across the two conditions. A Chi-square test revealed no significant association between profile and condition. χ^2^(2, *N* = 56) = 0.282, *p* = 0.87, indicating that the two variables are independent.

#### 8.9.2 Rapport

**Figure 8:**
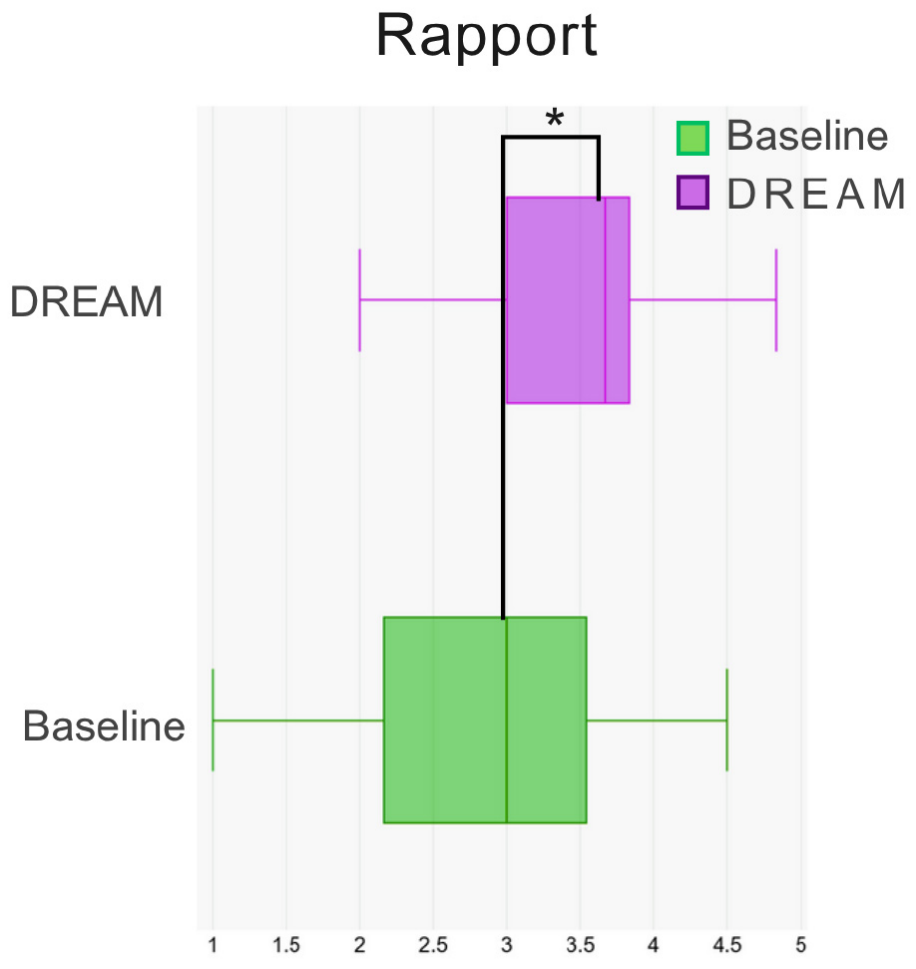
Average Rapport scores across conditions (*p < 0.05). The box plot shows the median and first and third quartiles. The whiskers show the min and the max.

The rapport scores are shown in Figure 8.

We performed a two-sample t-test to compare rapport scores between conditions. In both conditions, Shapiro-Wilk tests indicated that the normality assumption was met (Baseline: *p* =.35, DREAM: *p* = 0.44).

The t-test revealed a significant difference in rapport scores between the Baseline condition (M = 2.95, SD = 0.93) and the DREAM condition (M = 3.42, SD = 0.70), *t*(56) = 2.15, *p* = 0.04. This suggests that participants in the DREAM condition reported significantly higher rapport with the agent. The effect size was medium, *d* = 0.58. Therefore, participants in the DREAM condition seem to build more rapport with the agent than participants in the Baseline condition.

### 8.10 CEMI score

**Figure 9:**
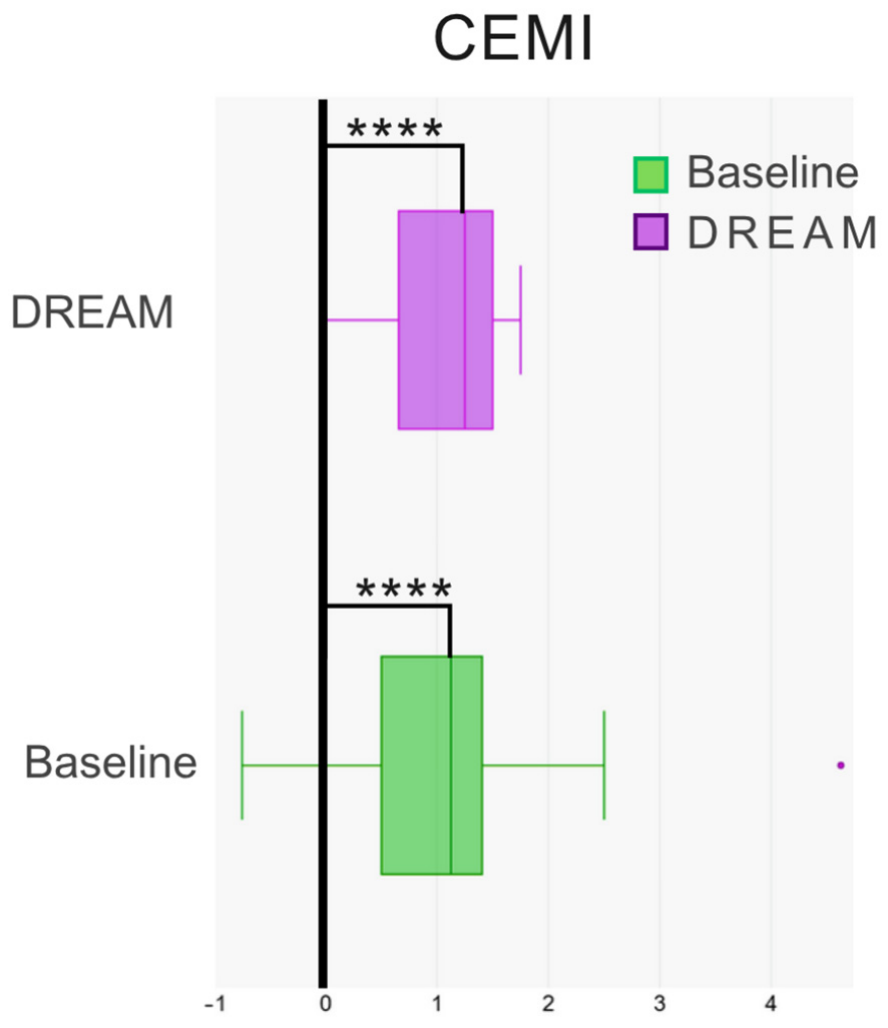
Average CEMI scores across conditions (****p < 0.0001). The black bold line represents the therapeutic threshold. The box plot shows the median and first and third quartiles. The whiskers show the min and the max.

The CEMI scores are shown in Figure 9.

The normality assumption was assessed using the Shapiro-Wilk Test. In the Baseline condition, CEMI scores were normally distributed (*p* = 0.64). However, normality could not be assumed in the DREAM condition (*p* = 0.04); therefore, a non-parametric Mann-Whitney U test was used.

The Mann-Whitney U test revealed no significant difference in CEMI scores between the Baseline and DREAM conditions, *U* = 432.5, *p* = 0.51. Therefore, there is no statistical difference in perceived MI quality between the Baseline and the DREAM condition. Nonetheless, for both the Baseline and DREAM conditions, CEMI scores were significantly above the therapeutic threshold (CEMI = 0).

For the Baseline condition, a one-sample t-test showed that the average CEMI score (M = 1.07, SD = 0.98) was significantly higher than the threshold: *t*(29) = 5.89, *p* = 0.000002, Cohen's *d* = 1.1, indicating a large effect.

For the DREAM condition, the average CEMI score (M = 1.02, SD = 0.55) was also significantly higher than the threshold: *t*(26) = 9.71, *p* < 0.0000001, Cohen's *d* = 1.84, again indicating a large effect. Therefore, the DREAM condition produces MI dialogues of quality perceived significantly higher than the therapeutic threshold.

### 8.11 DBS score

**Figure 10:**
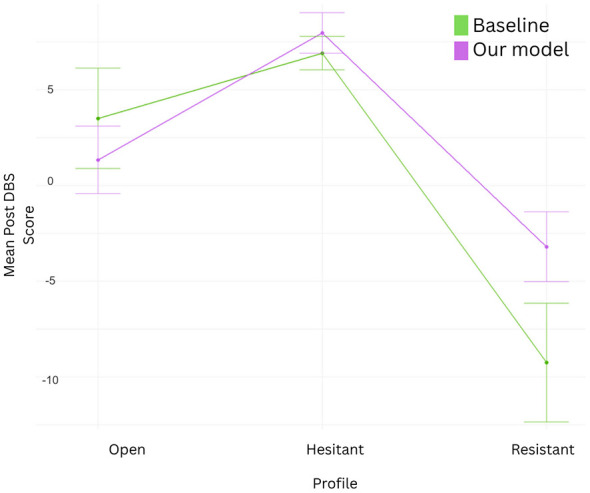
DBS score separated per condition and participant types.

The post-intervention DBS scores, adjusted for pre-intervention DBS scores, are illustrated in Figure 10.

As shown in Figure 10, the interaction plot suggests a possible interaction effect. To examine this further, an ANCOVA was conducted. One outlier was identified and removed before analysis.

A two-way analysis of covariance (ANCOVA) was conducted to examine the effects of Condition and Profile on post-intervention DBS scores, while controlling for pre-intervention DBS scores. The covariate (pre-intervention DBS score) was significantly related to the post-intervention DBS score, *F*(1, 49) = 19.35, *p* =.000059.

There was a significant main effect of the users' profile, *F*(2, 49) = 45.85, *p* < .0000001. The main effect of Condition was not statistically significant, *F*(1, 49) = 0.842, *p* =.36.

Importantly, there was a trend toward a significant interaction between Condition and users' profile, *F*(2, 49) = 2.02, *p* = 0.14, suggesting that the effect of users' profile may differ depending on Condition.

### 8.12 Discussion

This section discusses the findings of the experimental user study presented in Section 8.4 and compares these results with those obtained in Section 6 with the objective evaluation on the simulated environment.

#### 8.12.1 Adherence to MI principles

The first dimension assessed in the subjective study is adherence to MI principles. It is measured using the CEMI questionnaire, which evaluates participants' perceptions of the conversational strategies employed by the virtual counselor. The questionnaire focuses on the task-oriented aspect of the dialogue, specifically whether the agent demonstrates core MI behaviors such as empathy, collaboration, and support for autonomy.

The results indicate that both conditions, the plain LLM and the DREAM dialogue manager, achieved CEMI scores significantly above the therapeutic threshold. This suggests that participants perceived the agent's behavior as consistent with MI practices in both conditions. These findings validate the effectiveness of the prompts used for NLG, demonstrating that a properly designed prompt, even without a structured dialogue manager, can elicit MI-consistent responses from a plain LLM. However, the results also demonstrate that the DREAM manager is equally capable of maintaining MI adherence. This highlights the robustness of the DREAM framework, which maintains therapeutic quality while enabling greater transparency and adaptability within the interaction. Without the use of hierarchical reinforcement learning, the dialogue manager would focus on “Giving Solutions” as demonstrated in the objective evaluation in Section 6.1.2.

#### 8.12.2 Impact of hierarchical reinforcement learning

Participants reported significantly higher levels of rapport in the DREAM condition compared to the Baseline plain LLM. Rapport reflects the participant's emotional connection and sense of mutual understanding with the virtual counselor. It is especially important during the initial stages of the dialogue, where trust-building and engagement set the foundation for deeper therapeutic work or the *Engaging* phase.

This result from the experimental study aligns with the results obtained on the simulated environment from Section 6.1.2, which shows that the DREAM dialogue manager allocates more time to the *Engaging* phase at the beginning of the interaction than the baseline. In contrast, the plain LLM and the HRL ablation tended to quickly transition to task-oriented exchanges, potentially overlooking the value of early relational grounding.

These findings suggest that HRL, as implemented in the DREAM manager, plays a key role in structuring the dialogue in a way that mirrors the natural phases of MI. By explicitly modeling the dialogue flow at a higher level, distinguishing between *Engaging*, *Focusing*, *Evoking*, and *Planning*, HRL allows the system to dynamically allocate more time and strategies to rapport-building when needed. This structured approach contrasts with the decision-making of a plain LLM, which lacks an explicit mechanism to manage dialogue phases.

#### 8.12.3 Impact of meta-learning

The interaction effect observed in the DBS score between condition (DREAM vs Baseline) and user profile highlights the added value of meta-learning within the DREAM dialogue manager. While the overall main effect of the condition (DREAM vs Baseline) was not significant, the trend toward an interaction suggests that the DREAM system adapts its dialogue strategies based on the user's profile, whereas the baseline plain LLM does not show such adaptability.

Meta-learning helps the DREAM dialogue manager to adapt its behavior to the user's profile and the particular characteristics of that profile.

## 9 Conclusion and limitations

The goal of this study is to adapt LLMs at two complementary levels: to diverse user profiles and to the evolving sub-goals that structure dialogues. However, several limitations remain. A first limitation is the reliance on a theory-driven, manually specified reward function. While this approach ensures alignment with clinical MI protocols, it introduces subjectivity and exposes the system to potential reward hacking. Therefore, it remains unclear how sensitive the learned policies are to changes in reward weighting. Future studies should employ sensitivity analyses to mitigate this issue. Moreover, more principled approaches, such as inverse reinforcement learning, would be desirable but are currently impractical due to the scarcity of high-quality annotated MI dialogues. Second, the training process is constrained by data availability and computational cost: the user simulator is based on a Mistral LLM, which slows down interaction generation and forces compromises such as small batch sizes, few conversations per epoch, and overall limited training stability. Furthermore, due to the high computational cost of each training cycle, results are reported using a single seed; while evaluating multiple seeds would further strengthen the statistical robustness of the findings, it is currently computationally prohibitive. While additional epochs or a better reward function might further improve performance, these computational constraints limit the extent of exploration.

The use of a single-user LLM-based simulator, while validated, remains a limitation. This limitation is mitigated by our user study, which shows that the learned policy adapts to real humans and is well-received. However, the user study remains small and could be expanded in future work.

Another limitation concerns the phase detection mechanism, which currently relies on heuristics rather than expert-validated annotations. Incorporating labels from certified MI practitioners or using supervised models trained on clinical corpora would strengthen the evaluation of phase-level adaptation. Moreover, although the system can adapt to user profiles, it does so based on a pre-intervention assessment; online and continuous adaptation over multiple sessions for each user remains an open challenge (Araujo and Bol, 2025). Currently, the framework is designed for profile-based adaptation and does not explicitly adapt to entirely unseen user profiles. However, integrating a meta-learning architecture provides a scalable foundation for future enhancements. Specifically, the meta-learning framework could be extended to operate across multiple sessions. With additional training between sessions, the master policy could transition from a global profile adaptation to a personalization for each patient. Such longitudinal adaptation would enable the model to refine its strategy based on a patient's unique trajectory over time.

While LLMs provide strong generalization, they lack mechanisms to adapt to different user profiles and navigate complex dialogue phases. To overcome these limitations, we introduced a hybrid framework that couples an LLM with a hierarchical dialogue manager capable of modeling interaction phases and adjusting behavior to different user profiles. MI is a demanding application setting to evaluate this two-level adaptation, given its well-defined structure into phases and the need to tailor the interaction to the individual.

Our empirical results show that guiding an LLM through such a structured dialogue manager leads to more coherent conversational progression and improved user adaptation compared to a plain LLM baseline. Both objective metrics and subjective evaluations with human participants indicate enhanced rapport and better adherence to MI-relevant interaction patterns. Despite the reward function's limitations, training improved the system's ability to manage complex dialogues.

Finally, the current evaluation of the DREAM framework is limited to a single application domain (MI). While the architecture is theoretically generalizable to other structured dialogue tasks through the modification of the reward function, this remains to be empirically demonstrated. The modular nature of the HRL framework suggests it would be adaptable to other domains; for example, in a customer support context, the reward function could be formulated as a weighted combination of information acquisition, user engagement, and the provision of a technically accurate solution. In such a scenario, the HRL framework would infer and manage phases such as initial engagement, information gathering, and resolution, similar to the phase management demonstrated in the MI domain. Future research should explore this potential across a broader range of conversational tasks to further validate the framework's versatility.

Future study will also explore the use of alternative LLMs, more efficient simulators, improved reward formulations, and online meta-learning strategies to update the dialogue manager across long-term interactions. Finally, no safety layer currently filters or monitors model outputs, an essential component before deploying such systems in real-world settings involving sensitive user states.

Overall, this study demonstrates the value of hybrid architectures for guiding LLMs and highlights that achieving robust two-level adaptation requires both structural constraints and user modeling. While MI served as a testbed, the proposed framework could apply to a wide range of complex task-oriented or support-oriented dialogue systems that demand both goal sensitivity and personalization.

## Data Availability

The original contributions presented in the study are included in the article/Supplementary material, further inquiries can be directed to the corresponding author.

## Footnotes

1 Version of November 2024. https://huggingface.co/mistralai/Mistral-Nemo-Instruct-2407.

2
https://huggingface.co/nomic-ai/nomic-embed-text-v1.5
